# Article-level classification of scientific publications: A comparison of deep learning, direct citation and bibliographic coupling

**DOI:** 10.1371/journal.pone.0251493

**Published:** 2021-05-11

**Authors:** Maxime Rivest, Etienne Vignola-Gagné, Éric Archambault

**Affiliations:** 1 Science-Metrix Inc., Montréal, Québec, Canada; 2 Elsevier B.V., Amsterdam, Netherlands; 3 1science, Montréal, Québec, Canada; Algolia, FRANCE

## Abstract

Classification schemes for scientific activity and publications underpin a large swath of research evaluation practices at the organizational, governmental, and national levels. Several research classifications are currently in use, and they require continuous work as new classification techniques becomes available and as new research topics emerge. Convolutional neural networks, a subset of “deep learning” approaches, have recently offered novel and highly performant methods for classifying voluminous corpora of text. This article benchmarks a deep learning classification technique on more than 40 million scientific articles and on tens of thousands of scholarly journals. The comparison is performed against bibliographic coupling-, direct citation-, and manual-based classifications—the established and most widely used approaches in the field of bibliometrics, and by extension, in many science and innovation policy activities such as grant competition management. The results reveal that the performance of this first iteration of a deep learning approach is equivalent to the graph-based bibliometric approaches. All methods presented are also on par with manual classification. Somewhat surprisingly, no machine learning approaches were found to clearly outperform the simple label propagation approach that is direct citation. In conclusion, deep learning is promising because it performed just as well as the other approaches but has more flexibility to be further improved. For example, a deep neural network incorporating information from the citation network is likely to hold the key to an even better classification algorithm.

## Introduction

Bibliographic and bibliometric classifications of research activities and publications have a subtle but pervasive influence on research policy and on performance assessments of all kinds [1, 2]. In bibliometric assessments, assigning a research group’s or institution’s output to one field of research rather than another may drastically alter the results of its evaluation. The classification scheme and the design choices extend to the selection of reference groups and benchmark levels used for normalizations and comparisons. Briefly put, nearly all investigators and scholars whose research performance is evaluated with bibliometric indicators, or who have even a passing interest in the citation impact of their written works, are affected by design choices in some of the core classificatory systems of science that are commonly used in research evaluation. This explains why the development of relevant and precise classification systems carries so much weight.

Classifying documents is as old as libraries themselves. For instance, in the great Library of Alexandria, Callimachus classified work in tables called “Pinakes”, which contained the following subjects: rhetoric, law, epic, tragedy, comedy, lyric poetry, history, medicine, mathematics, natural science, and miscellanea [3]. Classifications are rarely consensual and are typically criticized shortly after inception, and this may have started as early as Aristophanes’s “pugnacious” criticism of Callimachus’s Pinakes [4].

A classification aims at grouping similar documents under a common class. Documents can share commonalities on various dimensions such as language, field, and so forth. The multiple dimensions of knowledge are pervasive in all characterization of research. As a consequence, classifications are required at various scales (such as at the journal level and the article level), and there are also various types of classes that can be used simultaneously to characterize research activities and publications (e.g., to reflect the organizational structure of universities or that of industry). For example, disciplines and faculties found in academia categorize themselves to reflect their topic of interest, as do Scopus All Science Journal Classification. Contrastingly, US National Library of Medicine’s Medical Subject Headings thesaurus rather aim to reflect a heterogenous mix of subjects and experimental methods.

All classifications of intellectual work present boundary challenges to various degrees (i.e., establishing clearly what is counted in and what is not). Classifying research documents such as journals and articles does not evade these challenges. In research, new knowledge is continuously created, and newer knowledge does not always fit snugly into pre-existing classes [5]. These characteristic challenges of classifying activities are true at all scales, even though some authors argue they are more particularly problematic at the journal level [6, 7]. The “fractal” nature of classification challenges means that scientific journals could frequently be classified in more than one class, but this is equally true of scientific articles. This gives rise to controversies over using mutually exclusive classification schemes, which make reporting more convenient and clearer, and multiple-class assignments, which are ontologically more robust.

One reason to use classifications in science studies and in research assessment is to capture, interpret, and discuss changes in research practices. Some level of abstraction and aggregation is useful to retrieve the higher-level trends and patterns that are most often the object of analyses conducted by institutions, government agencies, and all manner of commentators. For bibliometric evaluation, one of the main reasons to classify scientific work and literature is to normalize bibliometric indices. This is necessary because of disciplinary-specific practices in authorship and citation practices, as well as variations in citation patterns over time [8].

A defining aspect of classification systems, particularly those used in research evaluation, is the level of aggregation of the classification, such as at the journal or the article level, or at the conference or the presentation level. The classification of scientific work at the journal and article levels has been extensively studied [9–12]. Journal-level classifications often recapitulate historical disciplinary conventions and nomenclatures, making their use more intuitive for certain audiences, including research administrators, policymakers, and funders. They are certainly useful for journal publishers who need to categorize their journals and to present them on their websites in a compact manner, and they can also be used to specify the field of activity of authors [13].

Though there are many cases where journal-level classifications are useful, there are many cases where classifying articles is preferable and more precise. For instance, it is often useful in bibliometrics to individually classify each article in multidisciplinary/general journals such as *PLOS ONE*, *Nature*, or *Science*. Moreover, there are articles published in, for example, an oncology journal whose core topic could be more relevant to surgery. As a result, journal-level classifications are not tremendously precise compared to those at the article level [6, 8, 10, 11]. Without negating the need for journal-level classifications for many use cases, there are therefore several reasons to prefer an article-based classification to a journal-based one in a host of research evaluation contexts.

**Fig 1:**
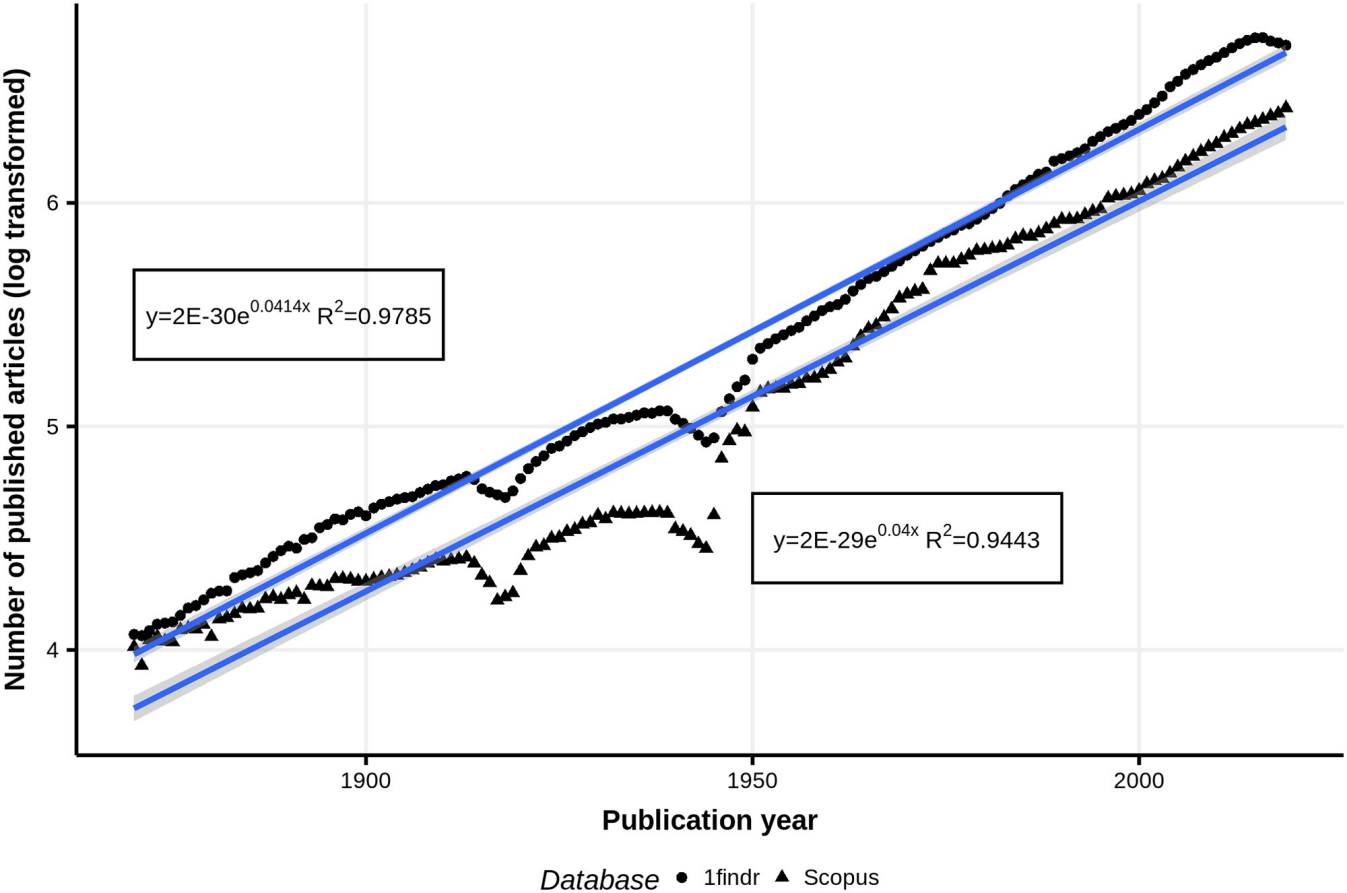
Growth of scientific articles published in scholarly journals, 1870–2015.

Knowledge is evolving extremely rapidly, and this creates notable classification problems at both the journal and the article levels. For instance, the *1findr* [14] database indexes the content of close to 100,000 active and inactive refereed scholarly journals, whereas the *Scopus database* [15] presents a more selective view of a similar corpus by selecting journals that are highly regarded by peers and/or that are the most highly cited in their fields (Fig 1). In both databases, the doubling period is approximately 17 years—meaning these indexes contain as many articles in the last 17 years as during all years prior. This rapid growth of scientific publications creates a huge strain on classification needs and not only because of the large number of articles that need to be classified every day. Furthermore, because of the evolving nature of scientific knowledge, new classes need to be added to classification schemes, which sometimes require overhauling the whole classification scheme and reclassifying thousands of journals and millions of articles. Performing this classification work manually is prohibitively expensive and time-consuming. This means that precise computational methods of classification are sorely needed.

Although articles have been manually classified by librarians for decades if not centuries, computational classification is a comparatively recent development given the requirement for processing capacity and large-scale capture and characterization of scientific publications [6]. Ongoing investigations carried out since the 1970s have resulted in the creation of a toolbox of classificatory approaches, each supported by varying bases of evidence, and from which investigators must pick and choose a technique based on their object’s features. Article-level computational classification techniques are mostly based on clustering algorithms and use a bottom-up approach, requiring relatively involved follow-up work to link the clusters obtained to categories that are intelligible to potential users. Determination of optimal levels of cluster aggregation is an ongoing development [11, 12, 16, 17]. Computational classification techniques have also benefited from advances in network analysis and text mining [18–21].

There is a plethora of methods developed and routinely used in the computer science fields of natural language processing and, more recently, machine learning and artificial intelligence (AI). These methods could advantageously be used in bibliometrics. More specifically, deep learning has recently been found to be extremely performant in finding patterns in noisy data, providing the network can be trained on a large enough volume of data. Yet, despite all the work that has been conducted to date on computational classification methods, bibliometricians have yet to make sustained use of AI, and in particular deep learning methods, in their classificatory and clustering practices.

The BioASQ challenge is a notable example of recent use of deep learning to bibliometrics [22] related task, but BioASQ’s researcher and bibliometrician have so far explored the topic in parallel. The BioASQ challenge aim at promoting methodologies and systems for large-scale biomedical semantic indexing since 2012 by hosting a competition where team try to automatically assign pubmed’s mesh-terms to scholarly publication [23]. Perhaps surprinsingly, none of the 9182 articles published in journals with (scientomet* OR informetri* OR bibliometri*) in their name ever mentioned BioASQ in their title, abstract, or keywords and only one of those 9182 publications cite a BioASQ related publication (according to this search on Scopus: “SRCTITLE (scientomet* OR informetri* OR bibliometri*) AND REF (bioasq)”). This latter publication is not focused on the subject of text classification.

As just mentioned, most solution to the BioASQ challenge now use technics related to deep neural networks. Several were based on BERT and BioBERT [22]. In this paper, we have chosen to explore another type of deep neural network (namely character-based neural network) because we felt that our task was different from the BioASQ task and less suited to those architectures. For example, the granularity of the task is several levels of magnitude different, we are classifying 174 subfield instead of approximately 27,000 mesh-terms. Furthermore, our training set is extremely noisy (56% accuracy). This noise may seem like a bad experimental design but is an integral part of the task facing bibliometricians. Indeed, any reclassification system or strategy useful to creating both an article and a journal level classification will need to be robust to noisy training set as it is prohibitively expensive to gather an article level training set. The last difference is that we used a much bigger set of input data and some are less amenable to tokenisation. Indeed, defining tokens for affiliations and source citation or other very rare and specific jargon words seemed suboptimal. Nevertheless, these were useful hypothesis that helped us choose a model architecture but as they are untested hypotheses other architectures should be explored in future works.

Generally speaking, classifications are only as good as the measurement and validation systems used to put them to the test [6, 7]. To examine how precise a deep learning classification technique could be, the results of this approach were compared to two other methods frequently used to map science: bibliographic coupling and direct citation links. This examination was based on scores of citation concentration [6] and on agreement with a benchmark dataset classified manually.

In addition to the novelty of this experiment with the use of a deep learning technique, the computational experiment was conducted at a very large scale. Often due to computational limits, bibliographic coupling has previously tended to be computed on record samples rather than at the corpus level of bibliographic databases such as Scopus and the Web of Science. The present paper examines the use of deep learning with bibliographic coupling, essentially at the corpus level of the Scopus database. One advantage of the approach proposed in the present paper is that it can be used to classify articles, as well as journals through aggregation of the resulting information. Obtaining a single classification scheme at both scales enables direct comparisons of article-level and journal-level classifications, which has seldom been realized in prior studies [10]. Lastly, and more importantly, whereas a large sample of the research on classifications addresses the creation of new classifications, this paper examines whether computational classification techniques, including the use of AI, can be used advantageously to maintain existing classification schemes compared to the use of a manual method.

## Methods

### Empirical setting and input data

This paper uses Science-Metrix’s bibliometric-optimized version of the Scopus database to retrieve scientific article metadata records and their associated citation data. This implementation used the 8 May 2019 build of the Scopus database. Publication records from the following types of sources were used in the experiment: book series, conference proceedings, and journals. For book series and journals, only the document types articles, conference papers, reviews, and short surveys were kept. For conference proceedings, only those classified as articles, reviews, and conference papers were used. Additional filters were applied such as removing sources not having valid ISSNs. Overall, 41 million Scopus publication records were used in the experiment.

### Training set

Science-Metrix’s journal classification was used to seed the journal classes to which articles or journals would be assigned to test the three computational classification techniques. This classification scheme is available in more than 25 languages. It contains a core set of 15,000 journals and is freely available (see science-metrix.com/classification and Supporting information). An additional set of some 15,000 journals that had fewer than 30 articles each when the original classification was designed are also used internally. Journals are classified in a mutually exclusive manner into 176 scientific subfields, and these subfields are mapped to 22 fields, which themselves are mapped to six domains.

A first version of that classification tree was designed from the top down by examining and drawing best practices from the classifications used by the US National Science Foundation, the Web of Science (now Clarivate, Thomson Reuters at the time), the Australian Research Council, and the European Science Foundation and the OECD. Journals were assigned to categories seeded from these classifications and then run through several iterations of automated suggestions, drawing insight from direct citation, Latent Dirichlet Allocation [24], and mapping of departmental names. Each iteration was followed by manual corrections to the suggestions obtained with these signals [9].

Except for the manual addition of new journals in the production version of the classification, the original version of the classification has not been updated in the last 10 years. As a consequence, some journals may have drifted in terms of subfields. In addition, many of the journals would no doubt be classified more precisely if the coupling were performed again due to improved signals (10 more years of publications means substantially more references and citations).

A newer version of this classification is currently under development and provides greater granularity, having 330 subfields, 33 fields, and seven domains. Although it is not yet used in bibliometric production, it can be seen in the 1findr abstracting and indexing database (see 1findr.com). The techniques experimented with here have been used to develop this expanded version, which currently provides a class for more than 56,000 journals, with an equal amount yet to be classified and more than 130 million scholarly articles waiting to be classified.

### Article- and journal-level classification

In contrast to a large share of the papers on classification, which use clustering techniques to determine fields and topics using a bottom-up approach, this experiment maps signals obtained from three coupling techniques to the existing Science-Metrix classification. The coupling/linking is performed at the article level, whereas journal-level classes are determined by the most frequent subfield of the sum of articles in each journal.

### Benchmarking deep learning against two bibliometric classification techniques

In order to benchmark the result obtained by the more experimental deep learning technique (DL), articles and journals were also mapped to the Science-Metrix classification using two commonly used techniques in bibliometrics: bibliographic coupling (BC) and direct citation (DC).

DL was compared to the other classification techniques using concentration scores of the references in review articles (at both the article and the journal levels) and was also compared to assignations by bibliometrics experts who manually classified articles.

#### Deep learning: A modified character-based convolutional deep neural network

The classifier deployed a character-based (i.e., the alphabetical letters composing the articles’ text) convolutional deep neural network. From single letters, the model learned words and discovered features. This character-based approach has recently been developed by adapting computer vision strategies to classification and has been used on large datasets of texts such as those from Wikipedia-based ontologies or Yahoo! Answers comment logs [25]. It has been found to be extremely performant in finding patterns in noisy data.

The model performed best when it was given the following features: authors’ affiliations, names of journals referenced in the bibliography, titles of references, publication abstract, publication keywords, publication title, and classification of publication references. All features, except the classification of publication references, were truncated, but not padded, to a specific maximum length (Table 1) and concatenated into one long string of text, which was itself truncated to a maximum length of 3,000 characters, the length of the 98^th^ percentile when ordering the concatenated vectors per length.

**Table 1:** List of inputs to the character-based convolutional deep neural network.

Branch	Feature	Length
Text branch		
	Title	175
	Keywords	150
	Authors’ affiliations	450
	Abstract	1750
	Journal title of references	1000
	Article title of references	500
Subfield branch		
	Vector of classifications of a publication’s references	176

The order of this table reflects the items’ order in the model. The lengths represent the maximum allowable length for each feature. Each character was embedded into a one-hot encoded vector of length 68. One-hot encoding is defined as a vector filled with zeroes and ones only at the position assigned to the character. Table 2 presents an example of character embedding for the word “cab”.

**Table 2:** Illustration of character embedding.

	**c**	**a**	**b**
**a**	0	1	0
**b**	0	0	1
**c**	1	0	0

When encoding, the 26 letters of the English alphabet, the 10 Arabic numbers, several punctuation signs (e.g., "-,;.!?:’_/\|@#$%ˆ&*˜+ = <>()[]{}\), and the space character each occupied one position in the vector. Any character that was not in this list was encoded as a hash sign (i.e., #). The subfield was the only feature not fed to the model as raw text. Instead, the subfield information was encoded into a vector of the proportion of each subfield mentioned in the reference list.

**Fig 2:**
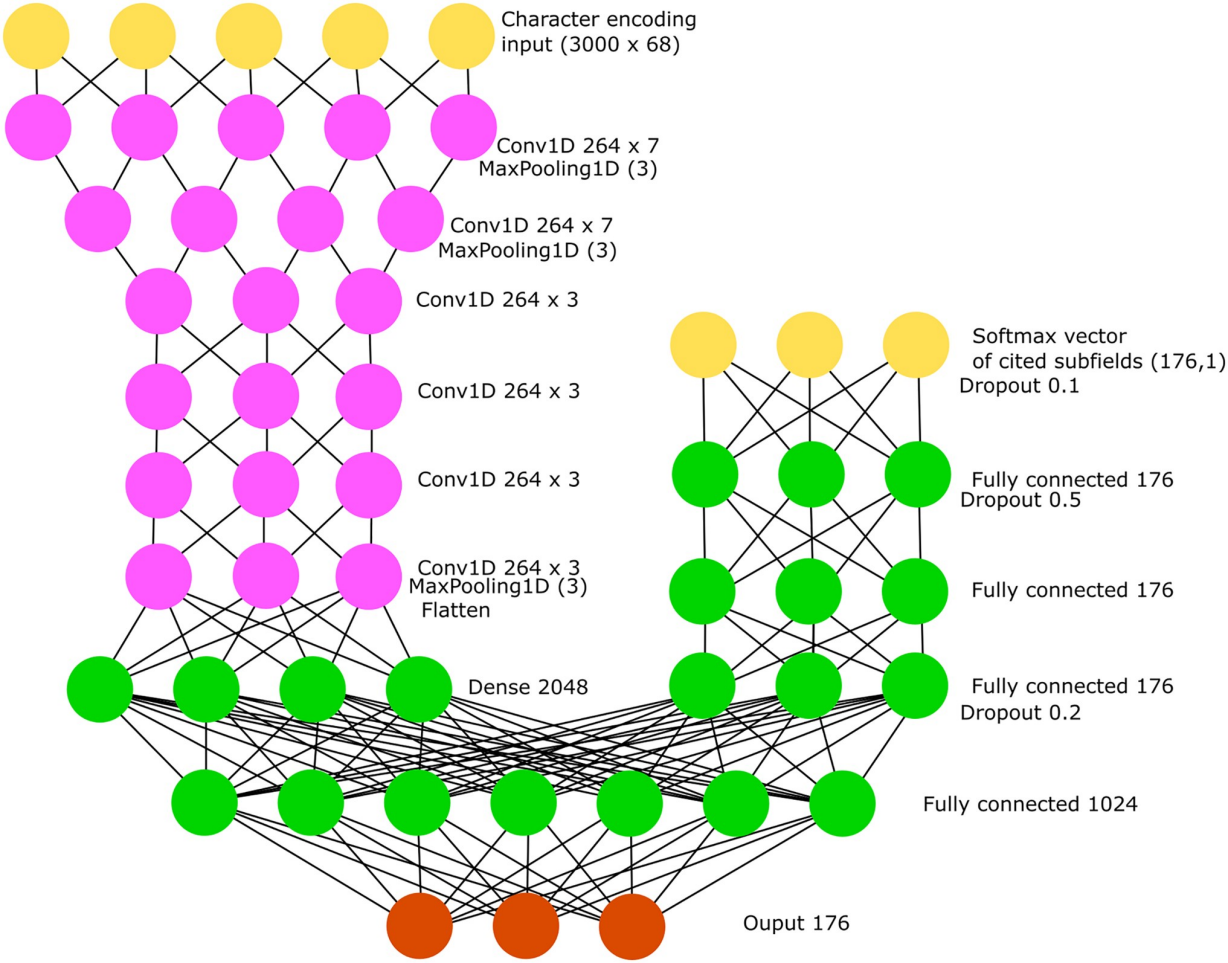
Deep neural network architecture.

The deep neural network architecture is shown in Fig 2. A rectified linear unit was used as the activation function between each layer, except after the last one, where a softmax was used instead. The kernels had a width of seven for the first two convolutions and three for the others. The model was trained with a stochastic gradient descent as the optimizer and categorical cross-entropy as the loss function. The gradient descent had a learning rate of 0.01, with a nesterov momentum of 0.9 and a decay of 0.000001. The model was trained on batches of 64 publications at a time and an epoch was considered passed after ~11,000 articles. The model was trained on a random sample of 24,000,000 articles.

#### Other machine learning algorithms

In addition to DL, three other machine learning approaches were considered for inclusion in the comparison to established bibliometric algorithm-based classifications. These are presented below but were all outperformed by the DL (the modified character-based convolutional deep neural network presented above) approach in the end. To keep the paper focused on the comparison between DL and bibliometric approaches, the rest of the paper will not present results for these three other methods after this section. However, the interested reader can find the results of our experiments in the (S1 and S2 Figs, S1 and S3 Tables).

A character-based neural network, without the above-mentioned modifications, was one of the methods tested. The model architecture followed the small ConvNets developed in Zhang et al. (preprint) (20), but a short description follows. The input layer was a character-encoded 2D matrix with 70 characters. Then, the input layer was fed into two consecutive layers of 256 kernels of width 7. Those two layers were each followed by a maxpooling layer of width equal to 3 and activated with a rectified linear unit. Then, four layers each of 256 features but this time of width 3 and without maxpooling followed the two initial layers of width 7. Then, the layer was flattened and passed to two fully connected layers of 1,024 nodes each, followed by a 0.5 dropout layer. Finally, after the last dropout we used a dense layer with as many nodes as there were subfields to predict. This last layer was softmax activated. A stochastic gradient descent with learning rate 0.01, a nesterov momentum of 0.9, and decay of 0.000001 was used as the optimizer. Mini batches were of size 64 and an epoch was considered passed (for the learning rate decay) after 11,000 articles were processed. The model was trained on a random sample of 24,000,000 articles.

A support vector machine (SVM) and a logistic regression were also tested as alternative shallow machine learning strategies. Both were based on term frequency–inverse document frequency (TF–IDF) one and two grams. The 150,000 tokens with the highest TF–IDF scores were used. The SVM and logistic regression models were trained on 1 million articles. There was no need to train on more articles since preliminary tests showed no model improvements after 0.75 million articles.

#### Bibliographic coupling

A BC-based similarity measure between each publication *P* and each subfield *S* was calculated and normalized as follows:

$${BCsimilarity}_{p,s}=\frac{\sum_{x\in X}log(x)}{T}$$

where *X* is the set of all BC values between publication *P* and all other publications of subfield *S*, and *T* is total number of citations given by all papers of subfield *S*.

#### Direct citation

A DC-based similarity measure between one publication *P* and one subfield *S* was calculated and normalized as follows:

$${DCsimilarity}_{p,s}=\frac{log(1+inCits)}{1+log(1+subfieldNR)}+\frac{log(1+outCits)}{1+log(1+paperNR)}$$

where *inCits* is the number of citations received by paper *P* from all papers in subfield *S*, *outCits* is the number of citations given by paper *P* to subfield *S*, *subfieldNR* is the total number of citations given by subfield *S*, and *paperNR* is the total number of citations given by paper *P*. In the end, paper *P* is classified in the subfield *S* with the highest *DCsimilarity*. This is calculated for all papers crossed with all subfields.

### Evaluation

Bibliometricians and machine learning researchers have different approaches to evaluate classification tasks. In bibliometrics, the task is often to search for the “true” classification, and no clear gold standard datasets exist for article-level classification of scientific publications. To mitigate the lack of a gold standard and in an attempt to quantitatively evaluate classification schemes, the concentration of reviews’ references (Herfindahl index) currently serves as the evaluation metric for such a task (more on that below) [6]. In machine learning research, it has become standard to evaluate classification methods by splitting the dataset into three sections: training, validation, and test. Unfortunately, this was not strictly possible here because the bibliometrics-based classification strategies (DC, BC) do not lend themselves well to such a dataset split. More importantly, the training set that we used here is only a rough approximation of what we want to achieve and not the ground truth per se. Indeed, the training set comprises articles roughly labeled by extending a journal’s label (from disciplinary journals) to all its articles, whereas our task is to classify each article independently of their journal classification. By doing so, we created a training set for which 56% of the articles corresponded to the mode classification of 5 experts. That said, we are still presenting those standard evaluation metrics in the supplementary material but we focus on two other evaluation strategies—namely, a human, manually assembled gold standard (which acts as our test set) and the Herfindahl index.

Performance measurements were produced for both article- and journal-level classification algorithms. We note already, however, that the bibliometric community is decisively moving toward article-level classification, where possible, for the reasons presented above, and that measurements for journal-based classification algorithms benefit from prior use of citation-based algorithms in the construction of the Science-Metrix ontology.

#### Comparison to training set

Precision, recall, specificity, sensitivity, and F1 were calculated. To measure the scores, the complete dataset was used (as opposed to a validation/test split); this was done to avoid biasing the results toward BC and DC as they are not amenable to the train/validation/test split. Moreover, in all machine learning cases, the training datasets were smaller than the validation dataset, which limits the confounding effect that overfitting could have. Furthermore, the training set is not ground truth, in fact only 56% of the articles corresponded to the mode classification of 5 experts. In other words, 44% of the labels in the training set were sub optimally assigned. This limits greatly the relevance of such metrics. Thus, the results for those are presented in the supplementary materials. Comparison to training set is done mostly to follow academic standard of the machine learning discipline, but the reader should interpret the results from this evaluation with great caution because the training set has 44% of it’s label sub optimally assigned. No statistical test were used as we measured precision, recall, specificity, sensitivity, and F1 on the whole dataset relevant for this task (i.e., the population) as opposed to using a few random samples [26].

#### Benchmark 1 (B1): Citation concentration (Herfindahl index)

As proposed by Klavans and Boyack [6], a citation concentration score was calculated on all articles having 100 or more references that could successfully be classified (i.e., the cited journals were in the set of classified journals). This technique assumes that large review articles tend to present an exhaustive analysis of a phenomenon by summarizing content from a large volume of prior research in a single or a few research subfields and specialties. In other words, and everything being equal, review papers would inherently capture clusters of topically related publications and would therefore act as appropriate reference points for benchmarking degrees of disciplinary concentration. A more accurate classification algorithm should therefore lead to a larger Herfindahl index. This use of the Herfindahl index as an accuracy measure applied to bibliometric classification appears to have been a novel development by Boyack and Klavans [27, 28]. Citation distribution profiles have also been measured with the Herfindahl index in a small number of unrelated studies aiming to assess the concentration and direction of “citedness” (i.e., uptake) among large populations of peer-reviewed articles [29].

The Herfindahl index itself originates in the economic literature but has been applied to measure the concentration of citations within a corpus in the last decade [29]. It has also been applied as a measure of disciplinary diversity among citations. Klavans and Boyack’s application of the index to measure clustering performance, however, appears to us to be a novel development.

Herfindahl index scores were measured for 379,413 papers. Citation concentration by subfield was calculated with the Herfindahl index for DL, BC, and BC, at the article and journal levels.

#### Benchmark 2 (B2): Manual article classification

To create a test set, five bibliometric analysts were asked to manually classify the same set of 100 randomly sampled scientific publications from disciplinary journals, and six other analysts were asked to do the same for another set of 100 articles from multidisciplinary journals. The analysts were asked to classify the publications as best they could, using whichever information was at their disposal. Most analysts used search engines to acquire additional information about authors and their affiliation. Analysts could assign more than one subfield to a publication when they were uncertain, in which case they were asked to rank subfields by relevance.

For each of the three classification techniques, the percentage of agreement between the computational and the manual classification was computed (the subfield most often assigned as the first choice by manual classification was used as the benchmark; in case of a tie, all top subfields were kept and computed classes were given a point for matching one or the other).

## Results

A comparison between each classification strategy and the Science-Metrix classification (which served as the training dataset) provided a first proxy to evaluate the quality of a classification strategy. We see that DL tended to replicate Science-Metrix classification more than BC and DC, given its higher macro-averaged F1 score and much higher macro-averaged precision than those calculated for BC and DC (S3 Table and see S1 Table for precision per subfield). That said, all journal-level classifications strategies were closer to the Science-Metrix journal level classification than any article-level classification strategy (S3 Table), which can be easily explained by the fact that the Science-Metrix classification is itself at the journal level. A measurement of the pairs of the most frequently substituted subfields by article-level DL and DC revealed that these pairs were generally formed of topically similar subfields. For example, the subfield *Networking & telecommunications* was assigned to an article belonging to *Automobile design & engineering* (according to the journal-level Science-Metrix classification) 65% of the time by DL (DC = 59%), *Economic theory* was substituted with *Economics* 51% of the time (DC = 34%), *Mining & metallurgy* was confused with *Materials* 47% of the time for DL (DC = 23%), and *Horticulture* with *Plant biology & botany* 42% of the time for DL (DC = 39%) (see S2 Table for full results). Altogether, these results show that very different strategies can similarly approximate a journal classification. This first set of results should be interpreted with caution since the Science-Metrix classification was an imperfect (53% accurate) journal-level classification used as a training set in the absence of a gold standard to act as a ground truth.

**Fig 3:**
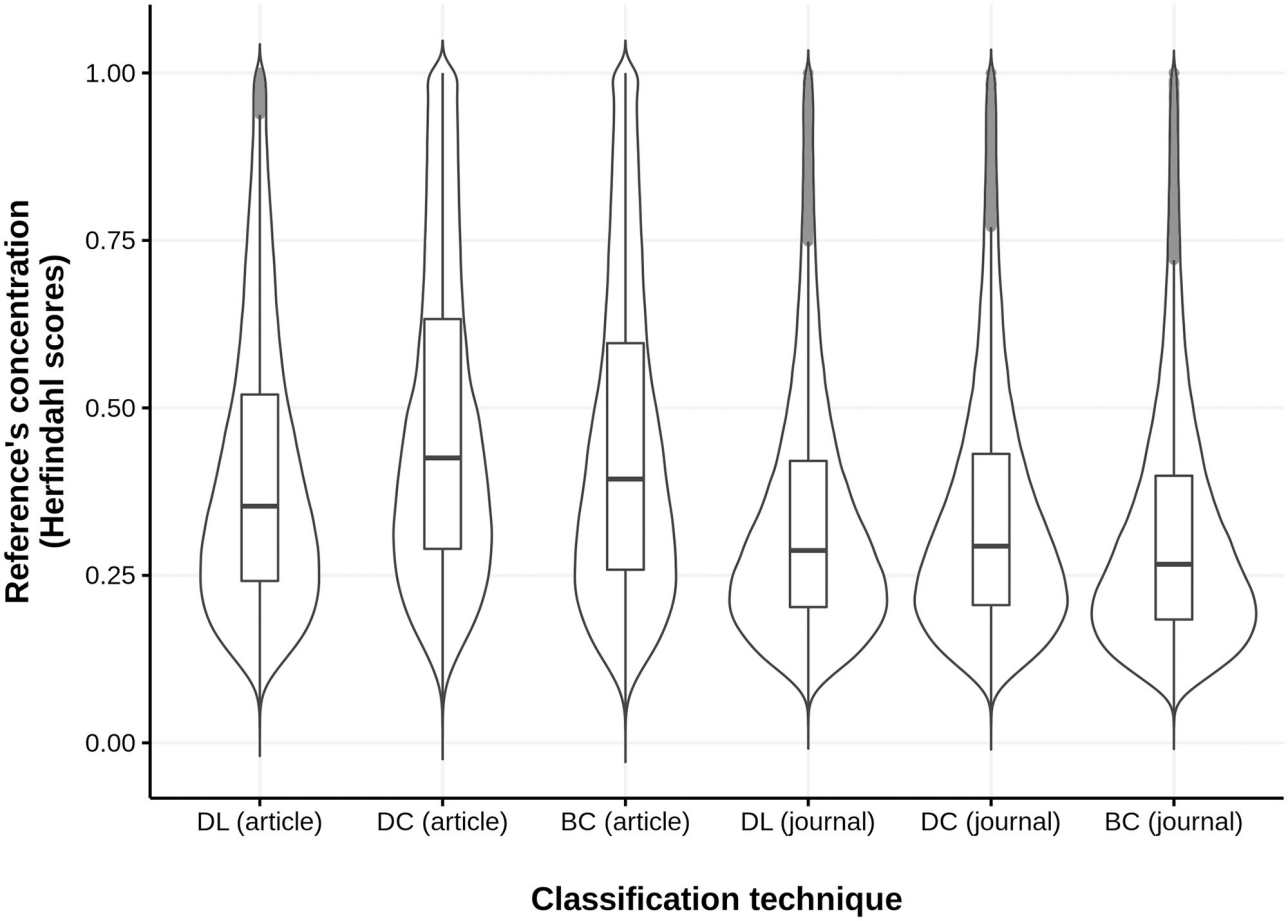
Herfindahl scores for three classification techniques at article and journal levels. The curves show a distribution of all the 379,413 Herfindahl scores that were computed for each classification technique, while the pale grey thicker vertical lines are outliers.

Median Herfindahl index scores obtained for the three classification techniques (B1) reveal that, though the scores are not all that markedly different, DL is the least effective technique for classifying articles (DL = 0.35 vs. 0.39 for BC and 0.43 for DC) and is fairly similar to DC when classifying journals (tie at 0.29 for DL and DC, with BC slightly lower at 0.27) (Fig 3). DL’s (article) median was the lowest of the three techniques because it had fewer high scores (as opposed to more low scores). This is shown in the width of the violins and the spread of the top boxes of the boxplots in Fig 3. More precisely, the width of the bottom parts of the violins are equivalent (as is the spread of the lower boxes), whereas the width of the top parts of the violins are narrowest at the top for DL.

**Fig 4:**
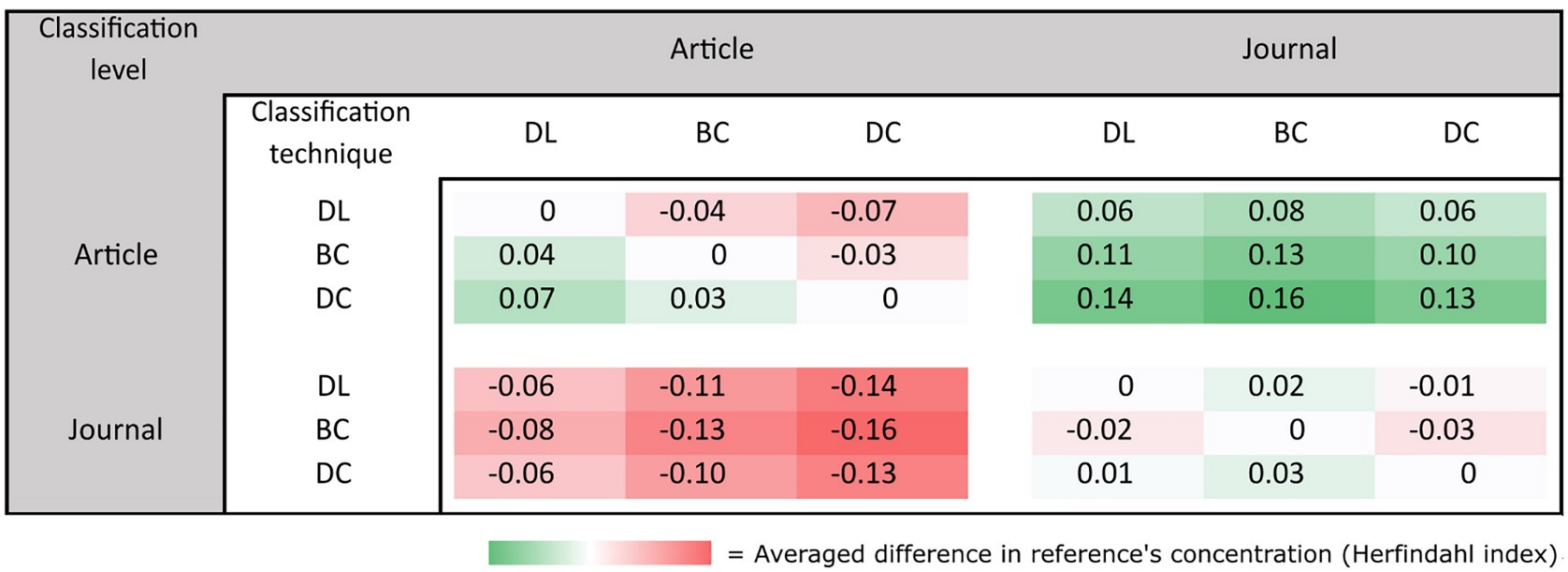
Article-level averaged differences between Herfindahl indexes of papers. The scores in rows are subtracted from those in columns, each time involving scores computed on all 379,413 papers having more than 100 matched references. A green cell indicates that the technique listed in the row yielded greater concentration than the technique listed in the column, and a red cell indicates the opposite.

Fig 4 shows the difference in Herfindahl scores between each technique, at both the article and journal levels. The scores in rows are subtracted from those in columns, each time involving scores computed on all 379,413 papers having more than 100 matched references. One can see that DL yielded a Herfindahl index score that was 0.04 lower than BC and 0.07 lower than DC at the article level. The results show that article-level classifications always provided better scores than any of these techniques at the journal level.

Benchmark 2 (B2) examines how computational techniques compare to manual classification. Usually, the subfields selected by experts were related to each other, but disagreement between experts was sometimes high. For example, for one document, two analysts chose the subfield biophysics, whereas others chose anatomy & morphology, bioinformatics, biomedical engineering, or evolutionary biology (Table 3). In one case, the five experts classified an article in five different subfields and in four different fields of science. Another scenario for disagreement was when a publication was interdisciplinary in its nature or applications (see the third document in Table 3). In contrast, article-level DC and BC consistently gave the same results, even in cases of overall disagreement among bibliometrics experts. In many cases, computationally obtained classifications tended to remain plausible, even in cases of divergence from the experts’ consensus.

**Table 3:** A sample of the gold standard.

Agreement between experts	Documents	Number of different experts’ first choices		Most mentioned subfield by experts		Classifications						
						Article level			Journal level			
		Subfields	Fields	Rank 1	Rank 2	DC	BC	DL	BC	DL	DC	SM
**lowest agreement**												
	Rabinovitch A, Aviram I, Gulko N, Ovsyshcher E. A model for the propagation of action potentials in non-uniformly excitable media. Journal of theoretical biology. 1999 Jan 21;196(2):141–54.	5	4	*biophysics	*anatomy & morphology	fluids & plasmas	fluids & plasmas	fluids & plasmas	bioinformatics	evolutionary biology	evolutionary biology	evolutionary biology
					*bioinformatics							
					*biomedical engineering							
					*evolutionary biology							
	Yamauchi S, Yamamoto N, Kinoshita Y. Improved stereoselective synthesis of optically active methylene lactone, key intermediate for the synthesis of 1, 2-oxidized furofuran lignan, by direct α-methylenation to butanolide. Bioscience, biotechnology, and biochemistry. 2000;64(10):2209–15.	4	3	*organic chemistry	*biochemistry & molecular biology	organic chemistry	organic chemistry	organic chemistry	biotechnology	biotechnology	biotechnology	biotechnology
					*biophysics							
					*biotechnology							
	Udhaya K, Sarala Devi KV, Sridhar J. Regression equation for estimation of length of humerus from its segments: A South Indian population study. Journal of Clinical and Diagnostic Research. 2011 Aug;5(4):783–6.	5	4	*anatomy & morphology	*archaeology	legal & forensic medicine	legal & forensic medicine	anatomy & morphology	dentistry	general & internal medicine	dentistry	pediatrics
					*general & internal medicine							
					*pediatrics							
					*public health							
**highest agreement**												
	Richards SN, Bryant JJ, Croom SM, Hopkins AM, Schaefer AL, Bland-Hawthorn J, Allen JT, Brough S, Cecil G, Cortese L, Fogarty LM. Erratum: the SAMI Galaxy Survey: can we trust aperture corrections to predict star formation?. Monthly Notices of the Royal Astronomical Society. 2016 May 11;458(2):1300-.	1	1	*astronomy & astrophysics		astronomy & astrophysics	astronomy & astrophysics	general physics	astronomy & astrophysics	astronomy & astrophysics	astronomy & astrophysics	astronomy & astrophysics
	Meister DW, Hearns KA, Carlson MG. Dorsal scaphoid subluxation on sagittal magnetic resonance imaging as a marker for scapholunate ligament tear. The Journal of Hand Surgery. 2017 Sep 1;42(9):717–21.	1	1	*orthopedics		orthopedics	orthopedics	orthopedics	orthopedics	orthopedics	orthopedics	orthopedics
	Gusakov EZ, Popov AY, Saveliev AN. Saturation of low-threshold two-plasmon parametric decay leading to excitation of one localized upper hybrid wave. Physics of Plasmas. 2018 Jun 1;25(6):062106.	1	1	*fluids & plasmas		fluids & plasmas	fluids & plasmas	fluids & plasmas	fluids & plasmas	fluids & plasmas	fluids & plasmas	fluids & plasmas

Three of the most and three of least agreed upon classifications are presented.

**Fig 5:**
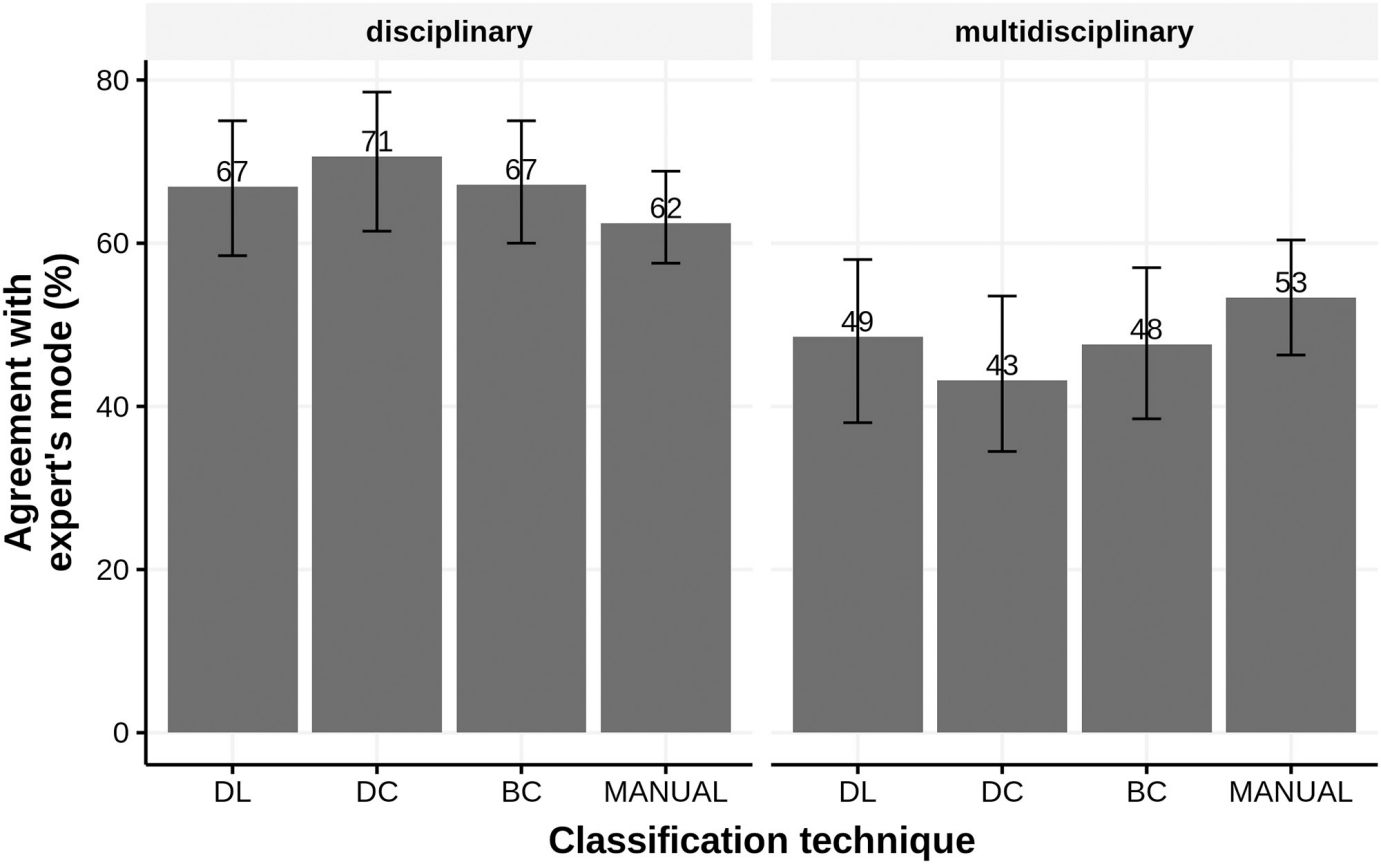
Agreement (%) between computational and manual article classification. Two datasets (disciplinary vs. multidisciplinary) of 100 papers were classified by five and six experts respectively to find the modal classification of each article.

Overall, B2 reveals that classification techniques were more accurate, in addition to having more similar scores between one another in the disciplinary dataset than in the multidisciplinary dataset (Fig 5). For both the disciplinary dataset and the multidisciplinary dataset, all computational classification techniques performed at a broadly similar level (i.e., all 95% confidence intervals overlapped).

## Discussion

Based on the examination of the differences obtained between each classification technique, considering the slight differences between these techniques, one can state that each technique is similarly effective overall to the two others. Though this suggests that classifying articles with DC, BC, or DL does not make a huge difference when mapping to an existing classification scheme, it is clear that it is always more precise for metrics to be based on an article-level assignment of classes, as shown previously [6].

Though the tests conducted here do not show DL to outperform other classification techniques overall it also worked just as well as BC and DC to classify articles published in disciplinary and multidisciplinary journals (B2). DL did not perform as well as BC and DC in the reference concentration test (B1) at both the article and journal levels, but it did not trail these techniques by a long measure either.

DC seemed to perform slightly better overall, yielding a high concentration of references in a subfield for review-type articles with large numbers of references (B1), and agreeing as much as others with experts (B2). DC and BC are well-established methods that have benefited from a decade of large-scale implementations and nearly five decades of experimentation at various scales. This paper shows that those strategies can be effectively applied to move from a journal to an article level classificationre for an existing taxonomy of science, subject to the existence of a seed dataset (the Science-Metrix classification was used to seed subfields to articles, based on the subfield of the journals in which they were published). This is quite different from the bottom-up, emergent clustering frequently done with those similarity metrics.

As multiple prior studies have reported, different classification methods are associated with trade-offs, and it may be difficult to single out one clearly superior approach [17, 20]. The findings of this experiment support the view that a toolset of classification approaches is available to deal with multiple sources of information, contexts, and goals and audiences for bibliometric work.

The techniques used in the present experiment made it possible to classify both articles and journals. This enables continuity with the classification schemes already used by stakeholders. As an example, the article-level DL algorithm presented here was recently used to improve the classification scheme underpinning bibliometric data production as part of the National Science Board’s Science and Engineering Indicators 2020 series of reports. The algorithm made it possible to redistribute articles from multidisciplinary journals within the 176 subfields of the Science-Metrix classification, in an effort that also involved achieving concordances with the National Science Foundation’s own Taxonomy of Disciplines.

Using the same classification scheme for articles and for journals has yielded results that further support the use of article-level classifications of science when computing metrics at the article level. Classifying articles on an individual basis yields more precise results than using journal-level classes at the article level, as practitioners often do when using the journal-based National Science Foundation classification or those offered in the Web of Science and Scopus.

DL presents the benefit of being able to classify articles even in the absence of reference/citation-related information, with more work needed to confirm this potential. DL also has the potential to aggregate features that were usually kept apart. As a first step toward that, this version of the DL model combined topical information (from the titles, keywords, and abstracts) with affiliation information (such as authors’ department names) and citation information (in the form of the distribution of classified references), and the appropriate weighting of those sources of information was tuned through the training of the model. It is conceivable that the Herfindahl index could be used in the creation of a loss function, which would let the model optimize that metric. Finally, there is nothing precluding the addition to the model of data obtained by BC and DC, and DL therefore appears to be highly promising as a solution to help classify both journals and articles in a cost-effective, big data production context.

## Conclusion

There has been recurring debate about the best methods for establishing and improving on the taxonomies and classifications of science used in the macro-organization of science, including for science policy planning, large-scale research assessments, and grant management. While bibliometric researchers tend to favor classifications developed with bottom-up clustering approaches, science policy bodies and funders still use and establish manually derived (i.e., reliant on expert judgment) rather than algorithmically derived classifications because of the continuity they offer with established institutional practices.

In this article, we presented a new approach to science classification that draws on a combination of deep learning approaches and bibliographic coupling that bridges article-level, bottom-up classifications of research work and predefined categories. The three approaches performed similarly to experts manual classification when task with creating an article-level classification from a journal-level classification. In addition, we hypothesize that the deep learning approach has high potential for further improvement and refinement going forward, notably by experimenting with graph neural networks to take advantage of the citation network and the author’s collaboration network, or by including the Herfindahl index in the loss function, or simply by better combining bibliographic coupling and direct citation with the modified character-based neural network presented here.

## Supporting information

S1 Fig Average difference in Herfindahl index including results from three other machine learning algorithms. (TIF)

S2 Fig Agreement (%) between computational and manual article classification including results from three other machine learning algorithms. (TIF)

S1 Table Precision per subfield for all classification strategy. (CSV)

S2 Table Top 3 most confused subfields per subfields for DL and DC. (CSV)

S3 Table Macro-averaged evaluation metrics including results from three other machine learning algorithms. (CSV)

S1 Dataset Manually classified scientific publications. (XLSX)

S2 Dataset Science-Metrix journal classification. (XLSM)
