# Supplementary figures and images for: Article-level classification of scientific publications: A comparison of deep learning, direct citation and bibliographic coupling
Source: PLoS One. 2021 May 11;16(5):e0251493. doi: 10.1371/journal.pone.0251493 (PMC8112690; doi:10.1371/journal.pone.0251493)

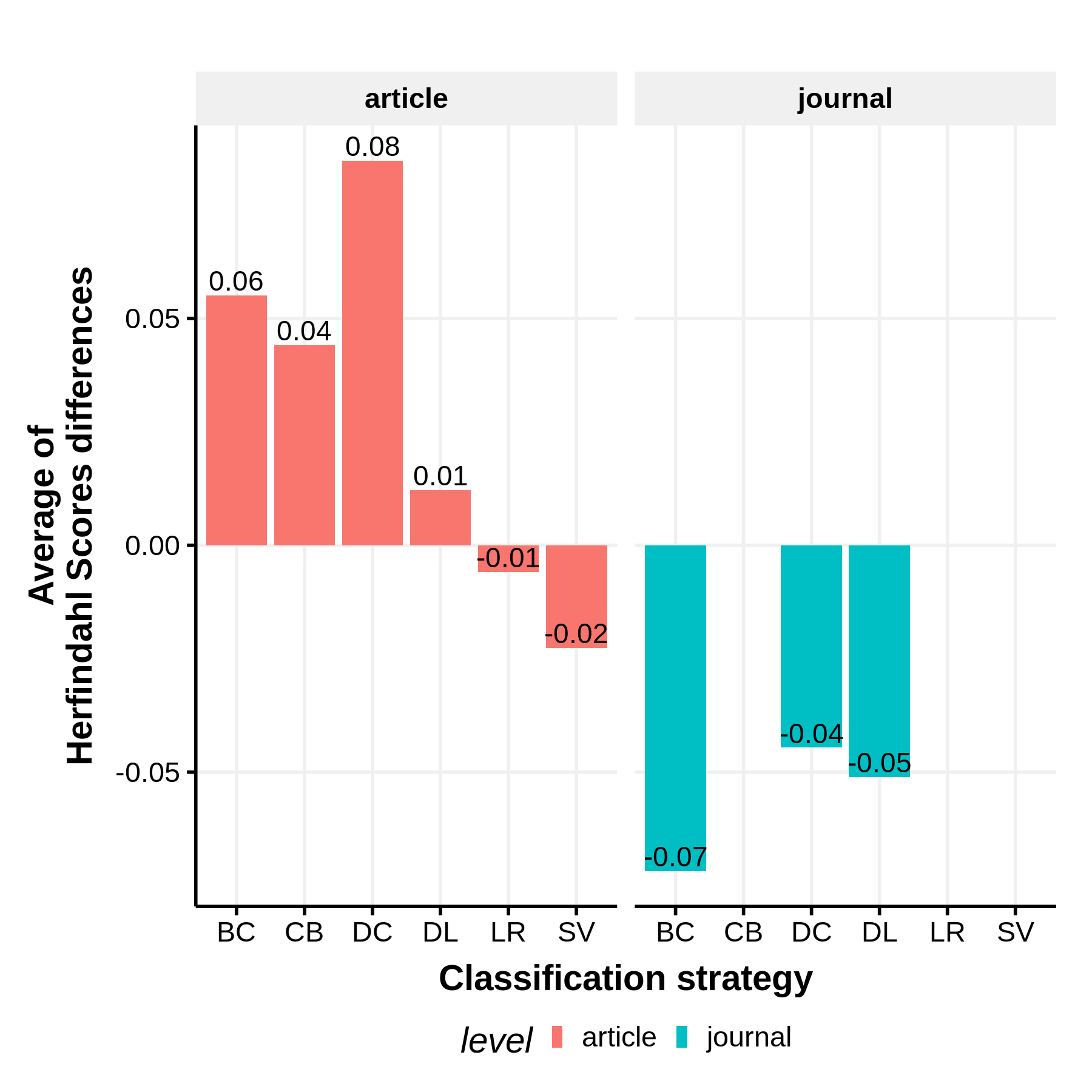

Supplement: S1 Fig — (TIF) [file pone.0251493.s001.tif]

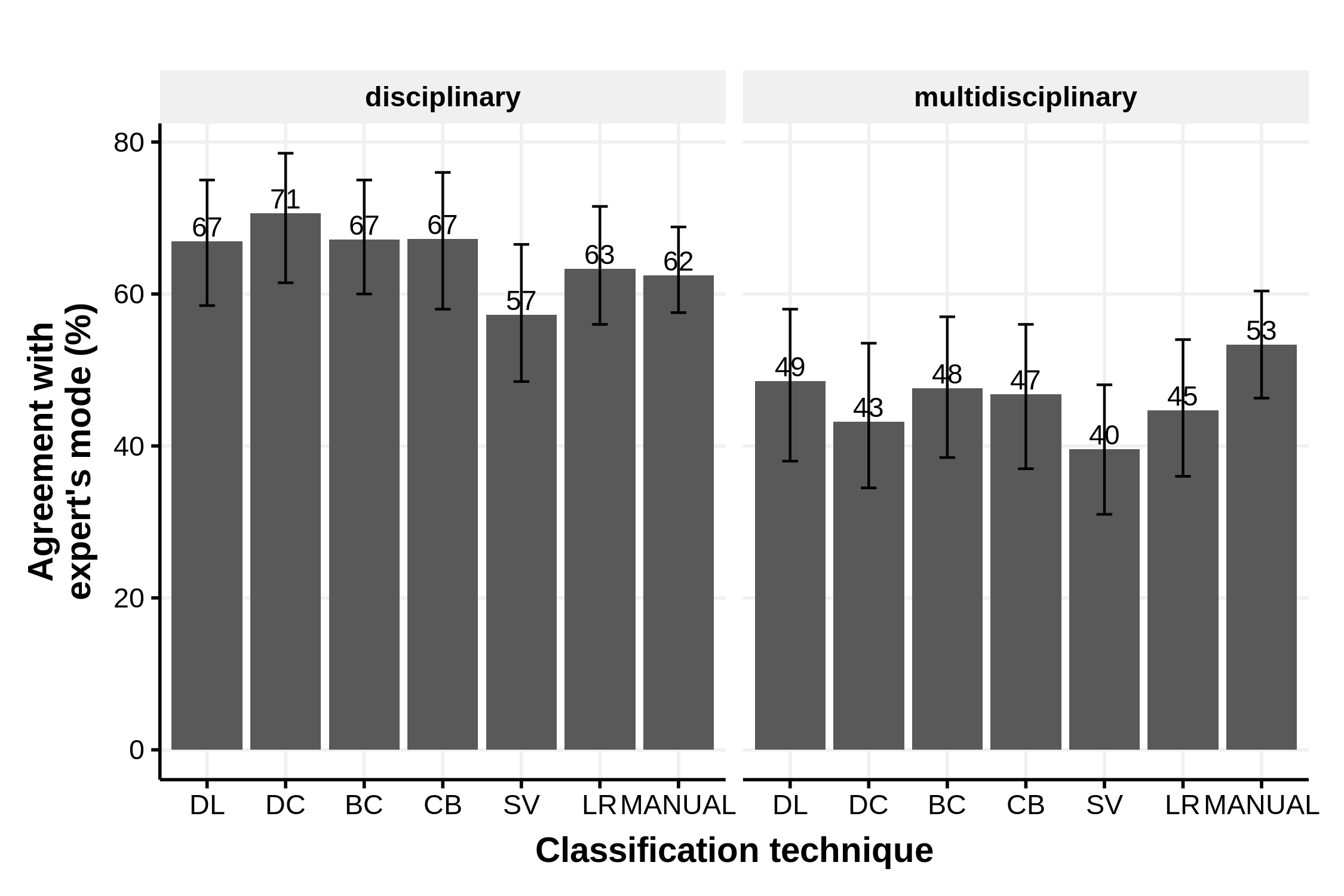

Supplement: S2 Fig — (TIF) [file pone.0251493.s002.tif]
